# Predictors of Unfavorable Outcomes in Patients with Atrial Fibrillation and Concomitant Heart Failure with Different Ejection Fractions: RIF-CHF Register One-Year Follow-Up

**DOI:** 10.1155/2019/1692104

**Published:** 2019-05-15

**Authors:** Igor Zhirov, Natalia Safronova, Yulia Osmolovskaya, Alina Alshevskaya, Andrey Moskalev, Sergey Tereshchenko

**Affiliations:** ^1^National Medical Research Center for Cardiology, Moscow, Russia; ^2^Biostatistics and Clinical Trials Center, Novosibirsk, Russia

## Abstract

**Background:**

Atrial fibrillation (AF) and heart failure (HF) are tightly interrelated. The concurrence of these pathologies can aggravate the pathological process. The geographic and ethnic characteristics of patients may significantly affect the efficacy of different types of therapy and patients' compliance. The objective of this study was to analyze how the features of the course of the diseases and management of HF + AF influence the clinical outcomes.

**Methods:**

The data of 1,003 patients from the first Russian register of patients with chronic heart failure and atrial fibrillation (RIF-CHF) were analyzed. The endpoints included hospitalization due to HF worsening, mortality, thromboembolic events, and hemorrhage. Predictors of unfavorable outcomes were analyzed separately for patients with HF and preserved ejection fraction (AF + HFpEF), midrange ejection fraction (AF + HFmrEF), and reduced ejection fraction (AF + HFrEF). Prevalence of HF + AF and compliance with long-term treatment of this pathology during one year were evaluated for each patient.

**Results:**

The study involved 39% AF + HFpEF patients, 15% AF + HFmrEF patients, and 46% AF + HFrEF patients. AF + HFpEF patients were significantly older than patients in two other groups (40.6% of patients were older than ≥75 years vs. 24.8%, respectively, *p* < 0.001) and had the lowest rate of prior myocardial infarctions (25.3% vs. 46.1%, *p* < 0.001) and the lowest adherence to rational therapy of HF (27.4% vs. 47.1%, *p* < 0.001). AF + HFmrEF patients had the highest percentage of cases of HF onset after AF (61.3% vs. 49.2% in other patient groups, *p*=0.021). Among patients with AF + HFrEF, there was the highest percentage of males (74.2% vs. 41% in other patient groups, *p* < 0.001) and the highest percentage of ever-smokers (51.9% vs. 29.4% in other patient groups, *p* < 0.001). A total of 57.2% of patients were rehospitalized for decompensation of chronic heart failure within one year; the risk was the highest for AF + HFmrEF patients (66%, *p*=0.017). Reduced ejection fraction was associated with the increased risk of cardiovascular mortality (15.5% vs. 5.4% in other patient groups, *p* < 0.001) rather than ischemic stroke (2.4% vs. 3%, *p*=0.776). Patients with AF + HFpEF had lower risk to achieve the combination point (stroke + IM + CV death) as compared to patients with AF + HFmrEF and AF + HFrEF (12.7% vs. 22% and 25.5%, *p* < 0.001). Regression logistic analysis revealed that factors such as demographic characteristics, disease severity, and administered treatment had different effects on the risk of unfavorable outcomes depending on ejection fraction group. The clinical features and symptoms were found to be significant risk factors of cardiovascular mortality in AF + HFmrEF, while therapy characteristics were not associated with it.

**Conclusions:**

Each group of patients with different ejection fractions is characterized by its own pattern of factors associated with the development of unfavorable outcomes. The demographic and clinical characteristics of patients with midrange ejection fraction demonstrate that these patients need to be studied as a separate cohort.

## 1. Introduction

Atrial fibrillation (AF), the most frequently encountered sustained cardiac arrhythmia, is the key risk factor for transient ischemic attack (TIA), stroke, and heart failure [1]. Its prevalence goes up with age, and population in Russia is aging nowadays. Heart failure (HF) is induced by structural and/or functional cardiac abnormalities and results in decreased cardiac pump function [2]. Atrial fibrillation and heart failure are closely interrelated [3, 4]. There currently is lack of understanding of the association between AF and heart failure with preserved ejection fraction (HFpEF); however, the clinical aspects seem to be rather important [5]. Comorbidity of atrial fibrillation and heart failure (AF + HF) is frequently observed because of the high prevalence of each disease entity, as well as due to shared risk factors and synergistic pathophysiology [6]. As the global population ages, the burden of both disease entities will be getting stronger over time [5]. Since these disease states have similar mechanisms, AF and HF tend to coexist [7–11]. One of the key features of their comorbidity is that each disease entity can trigger and aggravate the course of the other condition. The risk of thromboembolism in patients with atrial fibrillation becomes higher if they have such comorbidities as ischemic heart disease, hypertension, and diabetes [1]. The coexisting AF + HF increase the risk of thromboembolic complications, stroke in particular [1], and may deteriorate the cardiac function. In its turn, it results in aggravation of HF symptoms, thus leading to a self-perpetuating vicious circle [7]. Depending on whether AF or HF is the underlying condition, patient groups differ rather significantly in terms of outcomes and the required therapeutic approach. A patient who developed HF after AF has a more favorable prognosis [12] than a patient who had HF prior to AF [13, 14], probably because AF is a marker of severe course of the disease and can deteriorate the cardiac function [7]. Even this difference alone attests to significant heterogeneity observed in the total cohort of these patients. However, there are other important parameters subdividing patients with AF + HF into the fundamentally different groups.

According to the current clinical practice guidelines for managing patients with chronic heart failure (CHF) (2016), heart failure is divided into three clinical subtypes: HF with a preserved ejection fraction (HFpEF: EF ≥ 50%), HF with a midrange ejection fraction (HFmrEF: 40 ≤ EF < 49%), and HF with a reduced ejection fraction (HFrEF: EF < 40%) [7]. These patient cohorts strongly differ in terms of a number of parameters, from the epidemiology and etiology to the current severity and treatment strategies. Many questions regarding the differential treatment strategy are yet to be solved. One of the reasons is that our knowledge of HFpEF and HFmrEF is limited, and the data about these conditions have mainly been obtained from retrospective studies or post hoc analyses of randomized trials [7, 15]. Over the past 2 years, there has been a trend to conduct studies in isolated subgroups of patients having a certain ejection fraction [16–19]. This makes study groups more homogeneous and allows one to draw relevant conclusions. However, studies of this type are not devoid of drawbacks. The most fundamental one is that it is impossible to compare patients with different ejection fractions who are similar in nonmodifiable risk factors, such as area of residence, ethnicity, environment, climate, and family history. This fact prevents evaluation of the contribution of modifiable risk factors (lifestyle, dietary habits, current therapy, and prior therapy) to development and course of the disease. Simultaneous searching for intergroup similarities and differences needs to be conducted to understand the general principles of pathogenesis and the course of comorbid AF + AHF. One of the advantages of nationwide cohort studies as compared to narrowly focused prospective studies having strict inclusion criteria regarding the ejection fraction is that all three patient subtypes are included and can be compared.

There is a gap in understanding of the overall clinical pattern of the course of CHF + AF comorbidity and in selection of the optimal treatment strategies; so, it is very important to perform cross-sectional studies to fill this gap. The high prevalence of AF + CHF offers a unique opportunity for the researchers worldwide both to conduct large-scale prospective randomized clinical trials [20–27] and to collect the populationwide data using registries [1, 2, 28–31]. Each of these designs has its own advantages and drawbacks. Randomized clinical trials allow one to use the “refined” patient subgroups to answer a specific question regarding the treatment approach. Nation- and regionwide cohort studies make it possible to reveal the overall regularities in etiology and pathogenesis, as well as to demonstrate the real-world situation.

Our study was aimed to analyze how the features of the course of diseases and management of CHF + AF influence the clinical outcomes and collecting the data on compliance with clinical guidelines and on the long-term prevalence of this condition in Russia.

## 2. Patients and Methods

### 2.1. Study Design, Patient Selection, and Ethical Considerations

The analysis was conducted using the data retrieved from the Russian registry of patients with chronic heart failure and atrial fibrillation (RIF-CHF) that involved the data obtained in a multicenter prospective observational study in patients with CHF + AF (clinicaltrials.gov NCT02790801). Patients were recruited for survey participation at 30 medical centers in 21 provinces of the Russian Federation over the period between February 2015 and January 2016. The patients were selected on a competitive continuous basis. The planned total number of patients to be recruited was ≥1,000. The recruitment was stopped once 1,003 patients had been selected.

All patients had a confirmed diagnosis of CHF + AF comorbidity. The diagnosis of CHF was made according to the local Russian guidelines and corresponded to the ESC 2012 HF Guidelines criteria and ESC 2012 Update of the Guidelines for the Management of Atrial Fibrillation. The division into ejection fraction groups was conducted with allowance for the 2016 amendments to the Guidelines.

The inclusion criteria were as follows:

1. Age >18 years
2. Documented symptomatic chronic heart failure for at least 3 months prior to screening in accordance with the following criteria:
  - no additional laboratory verification of the diagnosis is needed at LVEF ≤40%
  - at LVEF >40%, the NT-proBNP level ≥300 pg/ml or BNP level ≥100 pg/ml
3. Hemodynamically stable nonvalvular atrial fibrillation

The exclusion criteria were as follows: transient ischemic attack within 3 days before enrollment; stroke during 14 days before enrollment; myocardial infarction within 14 days before enrollment; thromboembolic complications or thrombosis within 14 days before enrollment; heart failure because of valvular pathology; heart failure induced by infectious agents or infiltrative diseases; alcohol consumption; use of psychoactive drugs; peripartum heart disease; transient conditions; planned heart transplantation; implantation of biventricular pacemaker within 28 days before enrollment; any severe condition limiting patient's life to less than 3 months; HIV infection; alcohol consumption or intake of psychoactive drugs; participation in any experimental study within 30 days before enrollment; and patient being not ready to be contacted by telephone at the end of the follow-up period.

The survey was conducted in compliance with the Good Clinical Practice ensuring that the design, implementation, and communication of data are reliable; that patients' rights are protected; and that the integrity of subjects is maintained by the confidentiality of their data. The study was approved by the Local Ethics Committee of the Federal State Budget Scientific Institution “Research Institute of Cardiology” and registered at clinicaltrials.gov (no. NCT02790801). All patients provided written informed consent in accordance with the Declaration of Helsinki, which included their consent for their data to be analyzed and presented.

### 2.2. Data Collection: Baseline

Patients' life and past medical histories were collected at admission. Parameters to be collected and documented were as follows:

- General and demographic characteristics: date of birth, gender, educational status, occupation, residential region, smoking status, dietary habits, weight, height, and physical activity level
- Features and course of the disease in the past: dates when the patient was diagnosed with CHF and AF, predominant cardiac diagnosis, frequency of hospitalization over the past year, and past history of surgeries
- The current disease status: the reason for the current hospital admission (scheduled follow-up examination, decompensated heart failure, and surgical treatment), symptoms and signs, and blood pressure; instrumented test values (ECG, echocardiography); presence and severity of aortic and mitral regurgitation; and blood chemistry test
- Current treatment and patient's response to it: all drugs administered to treat CHF and AF; compliance with the treatment schedules according to the local and international guidelines; and efficacy of heart rate control for patients with permanent atrial fibrillation (maintaining the heart rate at < 100 bpm)
- Concomitant diseases
- Family history of hypertension, AF, and early-onset IHD

The clinical and laboratory data for each patient were collected 6 and 12 months after study enrollment.

### 2.3. Data Collection: Endpoints

The primary endpoint was hospitalization due to worsening of heart failure. HF hospitalization was defined as an overnight (or longer) stay in a hospital, with HF being the primary reason.

Secondary endpoints were death from a cardiovascular event (CV mortality), thromboembolic events (overall and for each category), and bleeding (ISTH major or CRNM). The time of event onset was documented for the endpoints. If possible, the follow-up was continued for 12 months.

### 2.4. Data Collection: Therapy Adherence

Therapy adherence was evaluated by a direct survey. At each of the three visits, patients were asked to provide information regarding the therapy they were currently receiving, as well as the therapy they had been receiving over the previous 6 months.

### 2.5. Statistical Analysis

Descriptive statistics were presented as absolute frequencies or medians with the interquartile range. The Mann–Whitney *U* test, or Pearson's *χ*2 test, or Fisher's exact test and nonparametric Kruskal–Wallis test by rank and median multiple comparisons were used depending on type of the data being processed. The Kaplan–Meier model was employed to analyze the achievement of target indicators. All the reported *p* values were based on two-tailed tests of significance; the *p* values < 0.05 were regarded as statistically significant. STATISTICA 7.0 software (StatSoft, USA) and RStudio software version 1.0.136 (Free Software Foundation, Inc., USA) with R packages version 3.3.1 (R Foundation for Statistical Computing, Austria) were used for the analyses.

## 3. Results

### 3.1. Baseline Characteristics

The survey included 1,003 patients with AF + HF. Almost half of them (46.5% of patients) had heart failure with reduced ejection fraction (AF-HFrEF); 38.6 and 15% of patients had heart failure with preserved (AF-HFpEF) and midrange (AF-HFmrEF) ejection fractions, respectively. The data on patient characteristics at baseline are summarized in Table 1–3.

Patients with preserved ejection function were significantly older (median age, 72 years (IQR 63 : 78) vs. 67 years (58 : 75) in the AF-HFmrEF group and 66 years (58 : 75) in the AF-HFrEF group). The percentage of women was the highest (65.4%) in the AF-HFpEF group and the lowest in the AF-HFrEF group (25.8%). Approximately 75% of patients with AF-HFpEF had never smoked, while only 50% of patients in the AF-HFmEF and AF-HFrEF groups were never-smokers (*p*=0.003).

**Figure 1:**
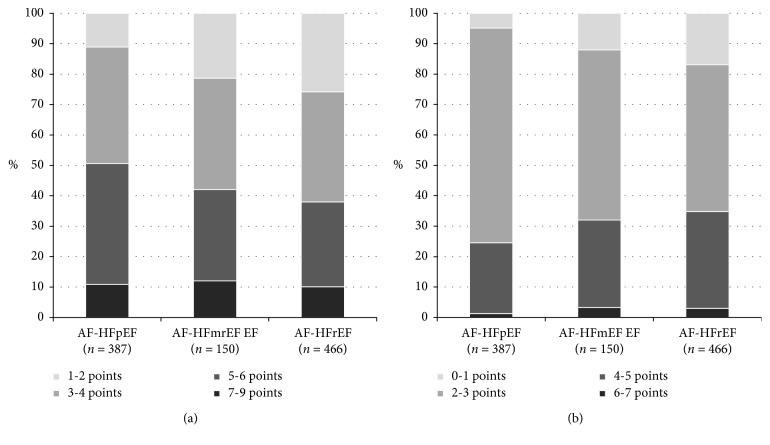
Risk assessment scales in studied subgroups. (a) CHA2DS2-VASc score. (b) HAS-BLED score.

The groups were comparable in terms of previous history of stroke (15, 14.7, and 16.7% in the AF-HFpEF, AF-HFmrEF, and AF-HFrEF groups, respectively; *p*=0.747) and significantly differed in terms of their history of infarction (25.3, 40.7, and 47.9%, respectively; *p* < 0.001). While in the AF-HFpEF and AF-HFrEF subgroups, patients developing AF already had CHF (50.9 and 47.9%, respectively), and the percentage of these patients in the AF-HFmrEF group was significantly lower (38.7%; *p*=0.039). CHF duration was comparable between the groups, while the oldest age of CHF onset was observed for the AF-HFpEF group (64 years; IQR 57.5 : 72.9). Regardless of the oldest age of AF onset (64.4 years; IQR 57.9 : 72.6), patients in the AF-HFpEF subgroup had the longest duration of AF (50 months; IQR 24 : 108). The percentage of patients with paroxysmal AF in the AF-HFpEF group was almost twice as high as that in the other two groups (37.2% vs. 20% in the AF-HFmrEF and 21.9% in the AF-HFrEF group). The rate control strategy was most effective in the AF-HFpEF group: the heart rate was >100 bpm only in 26.6% of patients (*p*=0.005 as compared to the other two groups). The groups differed in terms of risks of stroke and bleeding (Figure 1).

At baseline, the therapy differed significantly for patients in three subgroups (Table 4). Although the rate control strategy was prevailing in all the three groups, the percentage of patients treated using the rhythm control strategy was the lowest in the AF-HFpEF group (27.9%) and the highest in the AF-HFpEF group (40.6%) (*p* < 0.001). Chronic anticoagulant therapy was given to 79.3% of patients in the AF-HFmrEF group, 70.8% of patients in the AF-HFpEF group, and 63.3% of patients in the AF-HFrEF group (*p* < 0.001).

### 3.2. One-Year Follow-Up

**Figure 2:**
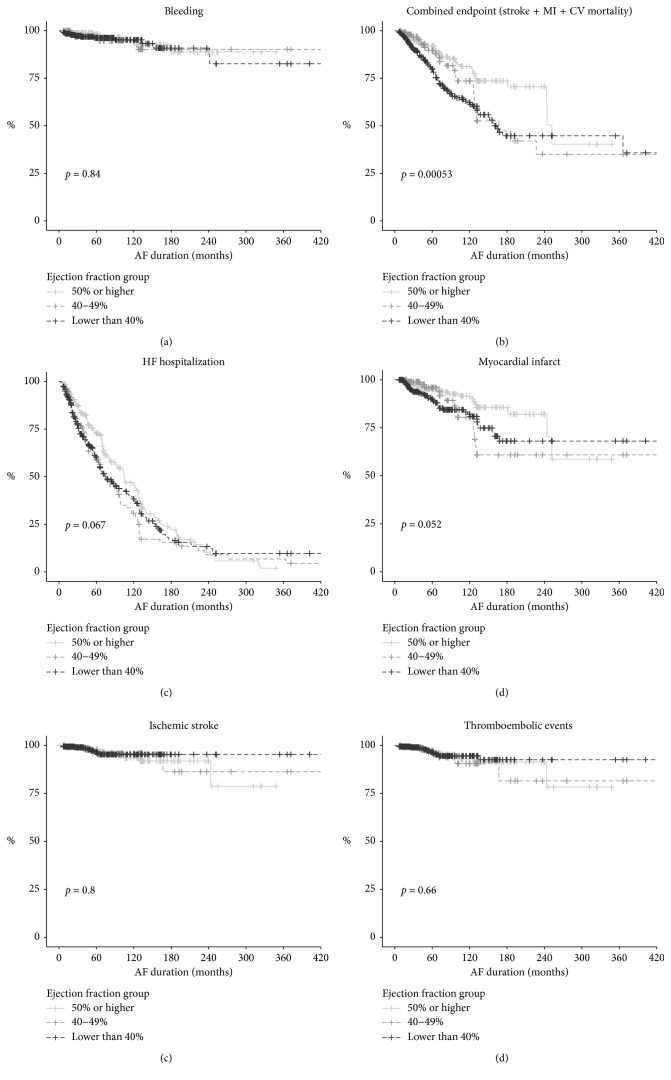
Kaplan–Meier curves for the studied subgroups.

During the one-year follow-up, 574 (57.2%) patients were hospitalized because of decompensated CHF at least once. The maximum frequency of hospitalization was observed for the AF-HFmrEF group (66%); the minimum, for the AF-HFpEF group (52.7%) (Table 5; Figure 2).

**Figure 3:**
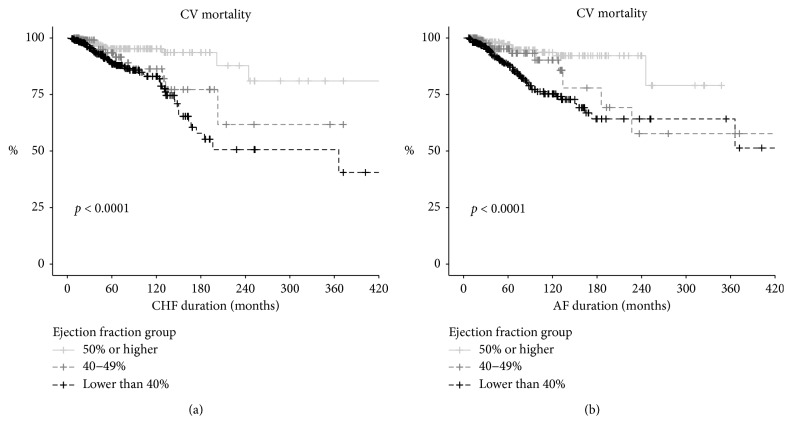
CV mortality probability.

In our cohort, the cardiovascular mortality rate went up as the EF decreased during the first year of follow-up: it was 4.1% in the AF-HFpEF group, 9.3% in the AF-HFmrEF group, and 15.5% in the AF-HFrEF group (*p* < 0.001). Significant variation in the dynamics of the mortality risk in groups depending on duration of AF and HF is shown in Figure 3.

The rate of all thromboembolic events in the total cohort was 3.4%; the rate of ischemic stroke was 2.7%. It is noteworthy that some patients (10 (1% of the total cohort)) experienced two different thromboembolic events (e.g., stroke and PATE) during the follow-up period.

The myocardial infarction rate in the total cohort was 101 (10.1%). Most of the infarctions were recurrent (96 out of 101). In patients with myocardial infarction experience, the rate of recurrent myocardial infarction was 25.1%, while the rate of MI among patients having no history of MI was as low as 0.8% (*p* < 0.001) during the follow-up. The significant variation in the myocardial infarction rate was also observed in the groups of patients with different ejection fractions: the AF-HFpEF patients were characterized by the lowest rate (6.7%).

A total of 39 cases of bleeding (3.9% of the total cohort) were documented during the follow-up: 13 (1.3%) cases of gastrointestinal bleeding, 6 (0.6%) cases of hemoptysis, 5 (0.5%) cases of intracranial hemorrhage, and 14 (1.5%) of cases of bleeding of other locations.

We conducted a search and analysis of factors for the primary and secondary points for three groups: AF-HFrEF, AF-HFmrEF, and AF-HFpEF. An analysis of factors significantly associated with outcomes led to the conclusion that the risk factors for adverse outcomes differ significantly for groups with different EFs (Table 6–8).

The number of specific signs of HF included pressure in the jugular veins, gallop rhythm, mixing the top of the jolt, wheezing in the lungs, and congestion in the lungs. Increase of venous pressure included jugular venous distention, pulmonary edema, pulmonary artery enlargement, and enlarged right heart. The number of typical symptoms of HF included dyspnea, fatigue, orthopnea, low effort tolerance, fatigue, edema, and apnoea. Bad habits included excess weight, diet, sedentary lifestyle, and hypercholesterolemia.

Among the demographic characteristics, gender was a significant predictor only in the HFpEF group. In this group, women were hospitalized significantly more often than men (79.6% vs. 35.1%). However, it is important to mention that the sex ratios in the groups differed significantly: women predominated in the HFpEF group, while only 25% of patients in the HFrEF group were female. Habits and lifestyle were significant for the HFrEF group. Alcohol consumption habits and physical activity level affected the hospitalization frequency. It is interesting that specific symptoms of heart failure were found to be significantly associated with the frequency of rehospitalization for patients with 40% < EF < 50%. However, they were not significant for patients with HFmrEF. The predictors related to echocardiographic and clinical characteristics of the cardiovascular status were subdivided into four groups. Presence and signs of persistent hypertension were significant both for the HFpEF and HFrEF subgroups. Signs of old myocardial infarction and concomitant surgeries were significant only for the HFrEF group. Signs of aortic, tricuspid, and pulmonic regurgitations were found to be predictors for HFpEF. Mitral valve dysfunction was the only factor being significant for patients with HFmrEF.

## 4. Discussion

The objective of our survey was to identify the features of the course and management of chronic heart failure in patients with atrial fibrillation and to collect the data on compliance with clinical guidelines and on long-term prevalence of this condition in Russia. The primary endpoint in our study was hospitalization because of decompensated heart failure. We demonstrated that the frequency of rehospitalization during the same year was 57.2%. Patients with HFmrEF were hospitalized significantly more often. The mortality, rates of thromboembolic events, and bleeding were selected as secondary endpoints. We found that reduced ejection fraction elevates the risk of cardiovascular mortality, while the risk of ischemic stroke does not increase. Using stroke, myocardial infarction, and CV mortality as a composite endpoint, we revealed that patients with HFpEF have significantly lower risks as compared to those with HFmrEF and HFrEF.

Several epidemiological studies of heart failure have been carried out in Russia. The largest ones are the EPOKHA-CHF (conducted more than a decade ago) [32, 33] and the TOPCAT study [17, 34, 35]. As reported in the EPOKHA-CHF epidemiological study, the prevalence of CHF in Russia is 11.7% of the overall population, and 56.8% of these patients have HFpEF. In our study, only 38.6% of patients had HFpEF. This fact demonstrates that the AF + HF comorbidity is characterized by more severe course of HF. In several U.S. community-based samples from 1990 to 2009, we observed divergent trends of decreasing HFrEF and increasing HFpEF incidence, with stable overall HF incidence and high risk for mortality [36]. In our study, the ratio between AF-HPrEF + AF-HPmrEF vs. AF-HPpEF groups is similar to the ratio in the American cohort. However, in the HPpEF group in our study, women predominate at a ratio of 2 to 1, and in the study by Tsao et al., there are more men by 20%.

We found that adherence to rational therapy of CHF as well as adherence to anticoagulant therapy is the key factors, reducing the risk of rehospitalization and cardiovascular mortality in patients with HFrEF. However, patients with preserved ejection fraction (HFpEF) were taking aldosterone antagonists almost twice less often. However, even in this group, therapy adherence was significantly higher than that in the Swedish Heart Failure Registry (SwedeHF) [37]. The data on using aldosterone antagonist therapy in AF + HFpEF patients are rather controversial. The TOPCAT trial involving 1,765 patients demonstrated that new-onset AF was not influenced by spironolactone. However, the meta-analysis by Neefs et al. [38] involving 5,332 patients with either existing or new-onset AF showed that aldosterone antagonist therapy reduced the risk of development or recurrence of AF. This conclusion was also valid for the AF + HF subgroup. Nevertheless, our findings indicate that patients with AF + HF comorbidity in Russia are prescribed aldosterone antagonist therapy regardless of ejection fraction. In this study, we found that aldosterone antagonist therapy in the HFrEF group significantly reduced the risk of rehospitalization because of subcompensated HF.

The HFmrEF group differed significantly from the other two groups with respect to the primary endpoint. In this group, the percentage of rehospitalized patients was substantially higher. We revealed that each EF group was characterized by its own factors associated with the primary endpoint.

Therapy received by the patients had a substantial effect on hospitalization frequency. For patients with HFrEF, the most important factor was whether or not they received anticoagulant therapy and its type. Rational therapy (RAS antagonist + beta-blocker + aldosterone blocker) substantially reduced the risk of rehospitalization. In the AF-CHF, prospective multicenter trial was found that beta-blockers were associated with significantly lower mortality but not hospitalizations in patients with HFrEF and AF, irrespective of the pattern or burden of AF. These results diverged from an individual patient-level meta-analysis conducted by Kotecha et al. [23], which included data from 10 randomized trials of beta-blockers versus placebo in HFrEF. Rienstra et al's main finding of meta-analysis indicates that the effect of beta-blockers in patients with HF and AF is significantly different from the effect of these drugs in patients with HF and sinus rhythm. Indeed, beta-blockers were not found to have a favorable effect on HF hospitalizations or mortality [26].

Therapy subtypes were found to significantly affect the mortality rate only in the HFrEF group. Similar to the primary endpoint, administration of novel oral anticoagulants, adherence to *β*-blocker therapy, and rational therapy of HF reduced the risk of mortality during the one-year follow-up.

Cardiovascular indicators were found to be significantly associated with mortality only for the HFpEF and HFrEF groups but not for HFmrEF patients. The only category of factors that was associated with mortality for all three HF groups included laboratory values attesting to renal insufficiency, anemia, and the BNP level. Furthermore, it was found that chronic kidney disease in HFmrEF patients abruptly increases the risk of mortality.

In our study, we revealed no intergroup differences in thromboembolism. By the time of study initiation, the mean risk of thromboembolism assessed using the CHA2DS2-VASc score was significantly higher only in the HFpEF group. This scale was a significant predictor of developing thromboembolism only for this group. An association between HF subtype and thromboembolic events was assessed under actual clinical conditions in the AF-HF substudy of the PREFER trial. The results demonstrated that the HF subtype predicts the residual risk of thromboembolism, and there is an inverse association between LVEF and hard thromboembolic endpoints (ischemic stroke and MACCE).

In this study, we revealed no intergroup difference in the bleeding rate. The low frequency of events did not allow us to analyze the risk factors for bleeding.

Our objective was to study the differences between the EF groups and to identify predictors of unfavorable outcomes using the real-world data. We investigated how the current clinical practice in Russia complies with the international guidelines. The revealed discrepancy was used for the analysis conducted, so that the Russian guidelines could be updated. Our study was the first one in this series and included patients from 23 Russian provinces.

This survey has a number of limitations. Despite the large pool of patients included in it, the groups being compared had some significant baseline differences because of population-wide features of Russian patients. Nevertheless, we have accumulated a large body of real-world data that show the current situation in clinical practice for the local data and can be compared to the data for patients from other countries.

## 5. Conclusions

All three ejection fraction subgroups are characterized by their own features of the course of AF + HF comorbidity; the risks of unfavorable outcomes differ for these subgroups. Reduced ejection fraction increases the risk of cardiovascular mortality but not the risk of thromboembolic events (such as stroke and systemic embolism). Rational therapy of CHF and adherence to anticoagulant therapy are the key factors reducing the risk of rehospitalization and cardiovascular mortality in patients with HFrEF. The rate control strategy has some advantages related to hospitalization because of decompensated CHF for patients with HFrEF, while the rhythm control strategy is beneficial for patients with HFpEF.

## Figures and Tables

**Table 1:** Demographic and anamnestic characteristics of patients included in the study.

Parameters	Total cohort (*n*=1003)	AF-HFpEF (*n*=387)	AF-HFmEF (*n*=150)	AF-HFrEF (*n*=466)	*p* level
*Demography and habits*					
Age, years	**68 (60** **:** **76)**	72 (63 : 78)	67 (58 : 75)	66 (58 : 75)	**<0.001**
The proportion of patients ≥65 years of age	**589 (58.7%)**	270 (69.8%)	82 (54.7%)	237 (50.9%)	**<0.001**
The proportion of patients ≥75 years of age	**310 (30.9%)**	157 (40.6%)	38 (25.3%)	115 (24.7%)	**<0.001**
Female sex, *n* (%)	**437 (43.6%)**	253 (65.4%)	64 (42.7%)	120 (25.8%)	**<0.001**
Body mass index ≥30	**360 (35.9%)**	147 (38%)	62 (41.3%)	151 (32.4%)	0.076
Low level of physical activity (exercise less than 30 min/day, no more than 3 times/week)	**570 (56.8%)**	191 (49.4%)	96 (64%)	283 (60.7%)	**<0.001**
*Smoking*					
Never	**603 (60.1%)**	295 (76.2%)	84 (56%)	224 (48.1%)	**<0.001**
Quit smoking	**216 (21.5%)**	53 (13.7%)	38 (25.3%)	125 (26.8%)	
Active smoker	**184 (18.3%)**	39 (10.1%)	28 (18.7%)	117 (25.1%)	
*Comorbidities and pathologies*					
Hypertension, fact	**653 (65.1%)**	263 (68%)	108 (72%)	282 (60.5%)	**0.012**
Hypertension, duration, years	**14 (10** **:** **20)**	13 (10 : 20)	10 (7.5 : 20)	15 (10 : 20)	0.916
Ischemic heart disease	**686 (68.4%)**	271 (70%)	107 (71.3%)	308 (66.1%)	0.336
Diabetes mellitus	**247 (24.6%)**	89 (23%)	38 (25.3%)	120 (25.8%)	0.632
Previous stroke/TIA	158 (15.8%)	58 (15%)	22 (14.7%)	78 (16.7%)	0.747
Previous myocardial infarct	382 (38.1%)	98 (25.3%)	61 (40.7%)	223 (47.9%)	**<0.001**
Vascular disease	**502 (50%)**	157 (40.6%)	74 (49.3%)	271 (58.2%)	**<0.001**
Abnormal renal function	**145 (14.5%)**	45 (11.6%)	24 (16%)	76 (16.3%)	0.123
Abnormal liver function	**101 (10.1%)**	12 (3.1%)	20 (13.3%)	69 (14.8%)	**<0.001**
*Family history*					
Family history of coronary artery disease early development	**230 (22.9%)**	78 (20.2%)	43 (28.7%)	109 (23.4%)	0.106
Hypertension in relatives	**516 (51.4%)**	231 (59.7%)	84 (56%)	201 (43.1%)	**<0.001**

**Table 2:** Clinical characteristics of AF + HF severity.

Parameters	Total cohort (*n*=1003)	AF-HFpEF (*n*=387)	AF-HFmEF EF (*n*=150)	AF-HFrEF (*n*=466)	*p* level
CHF duration, months	40 (12 : 96)	48 (22.5 : 100)	36 (12 : 72)	48 (12 : 96)	0.265
AF duration, months	48 (15 : 96)	50 (24 : 108)	38 (12 : 89)	40 (12 : 96)	**0.042**
Age at AF debut	62 (54.25 : 70.7)	64.4 (57.9 : 72.6)	60.8 (50.88 : 70.22)	59.9 (51.5 : 68.55)	**<0.0001**
Age at HF debut	62.1 (54.7 : 70.1)	64 (57.5 : 72.9)	61.65 (54.15 : 70.3)	60.9 (52.9 : 67.8)	**<0.0001**
AF debuted after HF	478 (47.7%)	197 (50.9%)	58 (38.7%)	223 (47.9%)	**0.039**
*Type of AF*					
Paroxysmal	276 (27.5%)	144 (37.2%)	30 (20%)	102 (21.9%)	**<0.001**
Nonparoxysmal	727 (72.5%)	243 (62.8%)	120 (80%)	364 (78.1%)	
*Blood pressure*					
Systolic	130 (120 : 140)	140 (130 : 150)	130 (120 : 140)	120 (110 : 140)	**<0.0001**
Diastolic	80 (70 : 90)	80 (80 : 90)	80 (70 : 90)	80 (70 : 80)	**0.01**
*Heart rate*					
Rate (beats/min)	84 (70 : 100)	80 (68 : 90)	85.5 (75.25 : 90.75)	84 (75 : 97)	0.226
Rate > 100, *n* (%)	327 (32.6%)	103 (26.6%)	56 (37.3%)	168 (36.1%)	**0.005**
CHA2DS2-VASc score, Me (IQR)	4 (3 : 5)	5 (3 : 6)	4 (3 : 5)	4 (2 : 5)	**<0.001**
HAS-BLED, Me (IQR)	3 (2 : 4)	5 (3 : 6)	4 (3 : 5)	4 (2 : 5)	**<0.001**

**Table 3:** The results of laboratory tests and instrumental examinations of patients at the time of inclusion with the study.

Parameters	Total cohort (*n*=1003)	AF-HFpEF (*n*=387)	AF-HFmEF EF (*n*=150)	AF-HFrEF (*n*=466)	*p* level
Left ventricular ejection fraction, %	**40 (35** **:** **58)**	60 (55 : 65)	43 (40 : 46)	34 (29 : 37)	<0.0001
Aortic insufficiency	410 (50.9%)	166 (42.9%)	57 (38%)	187 (40.1%)	0.529
Mitral insufficiency	805 (80.3%)	310 (80.1%)	123 (82%)	372 (79.8%)	0.841
Pulmonary insufficiency	367 (36.6%)	135 (34.9%)	52 (34.7%)	180 (38.6%)	0.459
Tricuspidal insufficiency	766 (76.4%)	284 (73.4%)	121 (80.7%)	361 (77.5%)	0.153
Left ventricular end diastolic dimension, cm	5.6 (5 : 6.3)	5 (4.6 : 5.3)	5.9 (5.3 : 6.38)	6.2 (5.7 : 6.91)	**<0.0001**
Left ventricular end systolic dimension, cm	4.1 (3.2 : 5.05)	3.1 (3 : 3.6)	4.5 (4 : 5)	5 (4.5 : 5.7)	**<0.0001**
Cardiothoracic index (%)	57 (54 : 62)	56.5 (53 : 61)	60 (55 : 63)	57 (55 : 63)	0.086
Ventricular extrasystoles, total	122 (17 : 775.5)	40 (8 : 327.25)	79 (13 : 1163)	277 (78.5 : 1319)	**0.029**
*Laboratory analysis*					
BNP	300 (158.25 : 602.48)	245.5 (152.25 : 429.75)	317.5 (142.25 : 507.15)	490.5 (186.52 : 941.75)	**0.008**
NT-proBNР	536 (349.5 : 1085)	562 (425 : 968)	338 (327 : 353.5)	1484 (289 : 2866)	**0.01**
International normalised ratio	1.27 (1.04 : 2)	1.15 (1 : 1.67)	1.29 (1.1 : 1.9)	1.42 (1.1 : 2.08)	**0.019**
D-dimer	1.2 (0.35 : 4.75)	1.38 (0.22 : 109)	2 (0.24 : 187)	1.1 (0.49 : 1.65)	**0.048**

**Table 4:** Medications for AF and CHF in studied groups.

Parameters	Total cohort (*n*=1003)	AF-HFpEF (*n*=387)	AF-HFmEF EF (*n*=150)	AF-HFrEF (*n*=466)	*p* level
*Treatment strategy for AF*					
Rhythm control	339 (33.8%)	157 (40.6%)	52 (34.7%)	130 (27.9%)	**<0.001**
Rate control	664 (66.2%)	230 (59.4%)	98 (65.3%)	336 (72.1%)	
Rational therapy of CHF	396 (39.5%)	106 (27.4%)	78 (52%)	212 (45.5%)	**<0.001**
Beta-blocker	830 (82.8%)	301 (77.8%)	136 (90.7%)	393 (84.3%)	**<0.001**
Antiarrhythmic	255 (25.4%)	123 (31.8%)	37 (24.7%)	95 (20.4%)	**<0.001**
RAS antagonist	218 (21.7%)	116 (30%)	27 (18%)	75 (16.1%)	**<0.001**
Aldosterone blocker	642 (64%)	164 (42.4%)	116 (77.3%)	362 (77.7%)	**<0.001**
Statin	606 (60.4%)	252 (65.1%)	89 (59.3%)	265 (56.9%)	**0.046**
Diuretic	883 (88%)	332 (85.8%)	131 (87.3%)	420 (90.1%)	0.137
Digoxin	360 (35.9%)	101 (26.1%)	53 (35.3%)	206 (44.2%)	**<0.001**
Oral anticoagulant (warfarin or/and NOAC)	688 (68.6%)	274 (70.8%)	119 (79.3%)	295 (63.3%)	**<0.001**
Warfarin	403 (40.2%)	157 (40.6%)	66 (44%)	180 (38.6%)	0.491
NOAC	335 (33.4%)	140 (36.2%)	55 (36.7%)	140 (30%)	0.107
Oral antiplatelet, total	466 (46.5%)	177 (45.7%)	61 (40.7%)	228 (48.9%)	0.200

**Table 5:** Main outcomes in AF-CHF subgroups.

Endpoints	Total cohort (*n*=1003)	AF-HFpEF (*n*=387)	AF-HFmrEF EF (*n*=150)	AF-HFrEF (*n*=466)	*p* level
Hospitalization for worsening of heart failure	574 (57.2%)	204 (52.7%)	99 (66%)	271 (58.2%)	**0.017**
Cardiovascular mortality	102 (10.2%)	16 (4.1%)	14 (9.3%)	72 (15.5%)	**<0.001**
Thromboembolic events	34 (3.4%)	14 (3.6%)	7 (4.7%)	13 (2.8%)	0.451
Ischemic stroke	27 (2.7%)	12 (3.1%)	4 (2.7%)	11 (2.4%)	0.776
Myocardial infarct	101 (10.1%)	26 (6.7%)	20 (13.3%)	55 (11.8%)	**0.014**
Combined point (stroke, IM, and CV death)	201 (17%)	49 (12.7%)	33 (22%)	119 (25.5%)	**<0.001**
Bleeding rate	39 (3.9%)	15 (3.9%)	7 (4.7%)	17 (3.6%)	0.815

**Table 6:** Univariate regression logistic analysis of the risk of hospitalization due to decompensation of HF.

Group of factors	Factor	AF-HFpEF		AF-HFmrEF		AF-HFrEF	
		OR (2.5–97.5)	*p* value	OR (2.5–97.5)	*p* value	OR (2.5–97.5)	*p* value

Laboratory tests	LDH	1.007 (1.004–1.011)	<0.001				
	Total bilirubin	1.035 (1.005–1.07)	0.031				
	Kreatinin					1.008 (1.002–1.014)	0.01

Demography	Age > 65 years	2.329 (1.462–3.745)	<0.001			1.736 (1.17–2.584)	0.006
	Female gender	1.866 (1.198–2.921)	0.006				

Habits and lifestyle	Smoking (ever)	1.852 (1.073–3.236)	0.028				
	Bad habits	2.009 (1.107–3.723)	0.023				
	Alcohol usage history					1.37 (1.038–1.828)	0.028
	Level of physical activity			0.549 (0.274–1.081)	0.085	0.616 (0.399–0.944)	0.027

Symptoms and syndromes	The number of specific signs of HF	1.482 (1.146–1.946)	0.003				
	Increase of venous pressure					2.383 (1.02–5.847)	0.048
	The number of typical symptoms of HF					2.275 (1.1–4.844)	0.028

Concomitant diseases	Diabetes mellitus	1.733 (1.048–2.908)	0.034				

CV system characteristics	Arterial hypertension					2.347 (1.524–3.663)	<0.001
	Duration of arterial hypertension	1.03 (1.002–1.061)	0.044				
	Degree of tricuspid insufficiency	1.408 (1.027–1.949)	0.036				
	Degree of aortal insufficiency	1.721 (1.074–2.865)	0.028				
	Degree of pulmonary insufficiency	3.69 (1.46–10.87)	0.01				
	Mitral insufficiency					1.543 (0.937–2.54)	0.087
	Hemodynamically significant coronary artery stenosis					2.166 (1.276–3.8)	0.005
	Cardiothoracic index (%)					1.138 (1.047–1.244)	0.003
	Vascular disease	1.73 (1.126–2.673)	0.013				
	Prior stroke or TIA or thromboembolism	1.866 (1.198–2.921)	0.006				

Therapy	Regular use of antiarrhythmic drugs	0.622 (0.393–0.978)	0.041				
	Regular use of ACE inhibitors	0.582 (0.371–0.907)	0.017				
	Permanent AF therapy with calcium channel blockers	0.505 (0.311–0.812)	0.005				
	Regular use of angiotensin II receptor blocker	0.466 (0.288–0.745)	0.002			0.587 (0.331–1.01)	0.06
	Regular use of anticoagulants					0.389 (0.257–0.587)	<0.001
	Permanent AF therapy with beta-blockers					0.279 (0.152–0.496)	<0.001
	Rational therapy of HF					0.409 (0.271–0.611)	<0.001
	Regular use of aldosterone antagonist					0.584 (0.361–0.942)	0.027
	Regular use of NOAC					0.588 (0.377–0.907)	0.017
	Rate control strategy (versus rhythm control strategy)	1.779 (1.156–2.747)	0.009			0.283 (0.125–0.599)	0.001

AF/HF features	HF developed after AF debut	2.002 (1.049–3.879)	0.037				
	AF duration	1.005 (1.001–1.01)	0.022				
	HF duration					1.005 (1.002–1.009)	0.003
	EF					0.958 (0.922–0.995)	0.026
	Persistent form of AF (versus paroxysmal form)	0.464 (0.296–0.722	0.001			2.755 (1.451–5.405)	0.002

Scales and risks	CHA2DS2-VASc	1.393 (1.215–1.608)	<0.001	1.191 (0.981–1.46)	0.083	1.215 (1.089–1.359)	0.001
	HAS-BLED	1.461 (1.174–1.836)	0.001			1.196 (1.014–1.414)	0.035

**Table 7:** Univariate regression logistic analysis of the risk of cardiovascular mortality.

Group of factors	Factor	AF-HFpEF		AF-HFmrEF		AF-HFrEF	
		OR (2.5–97.5)	*p* value	OR (2.5–97.5)	*p* value	OR (2.5–97.5)	*p* value
Laboratory tests	Total cholesterol			0.515 (0.291–0.851)	0.014		
	International normalised ratio	2.825 (1.353–7.937)	0.013				
	NT-proBNР	1.001 (1–1.002)	0.055				

Habits and lifestyle	Balanced diet	0.355 (0.102–1.279)	0.099				

Symptoms and syndromes	Anemia	5.618 (1.799–16.667)	0.002	4.219 (1.156–14.286)	0.022		
	The number of specific signs of HF (pressure in the jugular veins, gallop rhythm, mixing the top of the jolt, wheezing in the lungs, and congestion in the lungs)	2.299 (1.441–3.676)	<0.001	1.567 (0.924–2.653)	0.089	1.497 (1.163–1.927)	0.002
	The number of typical symptoms of HF (dyspnea, fatigue, orthopnea, low effort tolerance, fatigue, edema, and apnoea)	1.961 (1.335–2.941)	0.001			1.346 (1.121–1.629)	0.002

Concomitant diseases	Endoscopy presence of erosive ulcerative lesions gastrointestinal mucosa					2.353 (0.951–5.464)	0.053
	Abnormal liver function			5.291 (1.425–18.519)	0.009		
	Renal disease	4.184 (1.245–12.5)	0.013				

CV system characteristics	Aortic valve insufficiency			2.907 (0.915–10.101)	0.075		
	Arterial hypertension					2 (1.089–3.891)	0.032
	The duration of arterial hypertension (years)	1.066 (1.017–1.116)	0.006				
	Cardiothoracic index (%)	1.597 (1.133–2.841)	0.036			1.161 (1.053–1.294)	0.004
	History of infarction and/or stroke	3.521 (1.222–11.494)	0.024				
	Degree of pulmonary insufficiency					2.725 (1.269–5.882)	0.009
	Right atrium enlargement					3.546 (1.235–14.925)	0.04
	Degree of tricuspid insufficiency					1.37 (0.983–1.908)	0.061
	Echocardiographic signs of myocardial infarct (cicatricial changes and local conduction disturbance zones)	3.636 (1.233–10.526)	0.016			1.957 (1.129–3.509)	0.02
	Enlarged pulmonary trunk					2.375 (1.224–4.608)	0.01
	Prior major bleeding	6.494 (2.174–19.231)	0.001	3.891 (1.073–13.158)	0.03		

Therapy	Regular use of anticoagulants					0.389 (0.225–0.666)	0.001
	Regular use of NOAC					0.42 (0.202–0.806)	0.013
	Regular use of peripheral vasodilators					4.587 (1.695–11.905)	0.002
	Regular use of statins	0.254 (0.083–0.724)	0.011			0.627 (0.366–1.08)	0.089
	Regular use of ACE inhibitors	0.22 (0.069–0.84)	0.015				
	Permanent AF therapy with beta-blockers					0.404 (0.213–0.791)	0.006
	Permanent AF therapy with calcium channel blockers	0.172 (0.009–0.872)	0.091				
	Rational therapy of HF					0.432 (0.238–0.757)	0.004

AF/HF features	HF developed after AF debut					0.463 (0.209–0.987)	0.05
	Age of AF debut					1.037 (1.011–1.067)	0.007
	Heart rate more than 100 beats per second			4.545 (0.917–33.333)	0.081		

Scales and risks	CHA2DS2-VASc	1.385 (1.029–1.869)	0.031			1.163 (1.01–1.342)	0.037
	HAS-BLED	2.105 (1.305–3.425)	0.002	1.938 (1.238–3.175)	0.005	1.37 (1.098–1.715)	0.006

**Table 8:** Univariate regression logistic analysis of the risk of myocardial infarction.

Group of factors	Factor	AF-HFpEF		AF-HFmrEF		AF-HFrEF	
		OR (2.5–97.5)	*p* value	OR (2.5–97.5)	*p* value	OR (2.5–97.5)	*p* value
Laboratory tests	LDH	1.006 (1.002–1.01)	0.004	1.007 (1.001–1.014)	0.046		
	Triglycerides					1.566 (1.11–2.362)	0.02

Demography	Age> 65 years	3.115 (1.044–13.398)	0.071	3.27 (1.099–12.063)	0.047		

Habits and lifestyle	Level of physical activity	0.455 (0.173–1.069)	0.086				
	Unbalanced diet					4.714 (1.363–29.721)	0.038

Symptoms and syndromes	Signs of arterial hypertension (accent of the second tone on the pulmonary artery and left ventricular hypertrophy)	3.242 (1.269–9.964)	0.022	10.108 (1.969–185.253)	0.027		
	Anemia					1.964 (0.84–4.21)	0.097
	The number of specific signs of HF (pressure in the jugular veins, gallop rhythm, mixing the top of the jolt, wheezing in the lungs, and congestion in the lungs)	1.87 (1.256–2.754)	0.002	1.962 (1.237–3.208)	0.005		

Concomitant diseases	Abnormal liver function			4.417 (1.34–13.764)	0.011		

CV system characteristics	Aortic valve insufficiency	0.418 (0.148–1.045)	0.082	7.368 (2.457–27.37)	0.001	3.427 (1.565–7.683)	0.002
	Vascular disease	8.226 (3.029–28.777)	<0.001				
	ECG abnormalities			10.88 (2.92–70.815)	0.002		
	History of infarction and/or stroke	9.643 (3.547–33.762)	<0.001				
	History of cardiomyopathy	1.591 (0.827–2.635)	0.096	1.68 (0.88–2.999)	0.086	1.528 (0.947–2.371)	0.068
	Pulmonary insufficiency	6.283 (2.553–17.746)	<0.001	2.632 (0.963–7.412)	0.06	0.499 (0.248–0.948)	0.041
	Right atrium enlargement			2.632 (0.963–7.412)	0.06	0.499 (0.248–0.948)	0.041
	Family history of early ischemic heart disease			0.256 (0.039–0.972)	0.08	1.911 (1.009–3.569)	0.044
	Hemodynamically significant coronary artery stenosis			3.316 (1.1–9.569)	0.028	2.036 (1.025–3.888)	0.035
	History of coronary arteries stenting	3.311 (1.131–8.591)	0.019	4.727 (1.528–14.174)	0.006	2.043 (0.99–4.011)	0.044
	Thromboembolism of pulmonary artery	5.873 (0.809–29.014)	0.041	5.7 (1.041–28.378)	0.032		
	Degree of tricuspid insufficiency					1.601 (1.105–2.323)	0.013
	Venous thrombosis of the lower extremities	4.543 (0.966–16.216)	0.03	9 (0.76–127.873)	0.078		
	Echocardiographic signs of myocardial infarct (cicatricial changes and local conduction disturbance zones)	9.509 (3.986–24.457)	<0.001	10.51 (2.822–68.395)	0.002	4.459 (2.144–10.482)	<0.001
	Enlarged pulmonary trunk	4.165 (1.47–11.651)	0.006	9.797 (2.931–39.334)	<0.001	2.727 (1.323–5.652)	0.006

Therapy	Regular use of rivaroxaban			0.114 (0.006–0.79)	0.057		
	Regular use of digoxin			0.324 (0.072–1.051)	0.088		
	Regular use of ACE inhibitors			0.407 (0.147–1.158)	0.084		
	Regular use of ivabradin					6.313 (1.516–24.687)	0.007

AF/HF features	HF developed after AF debut	3.154 (1.026–11.799)	0.058			0.471 (0.19–1.101)	0.089
	Age of HF debut	1.051 (1.005–1.101)	0.033	1.045 (0.996–1.102)	0.082		
	Persistent form of AF					0.158 (0.009–0.752)	0.071
	Heart rate at rest			0.382 (0.143–0.945)	0.043		

Scales and risks	CHA2DS2-VASc	1.372 (1.077–1.752)	0.01	1.398 (1.069–1.865)	0.017		
	HAS-BLED			1.609 (1.09–2.432)	0.019		

## Data Availability

The datasets generated during and/or analyzed during the current study are available from the corresponding author on reasonable request.
